# EAFormer: Edge-Aware Guided Adaptive Frequency-Navigator Network for Image Restoration

**DOI:** 10.3390/s25185912

**Published:** 2025-09-22

**Authors:** Wenjie Xie, Dong Zhou, Wenshuai Zhang, Wenrui Wang

**Affiliations:** Research Institute of Electronic Science and Technology, University of Electronic Science and Technology of China, Chengdu 611731, China; 202322230102@std.uestc.edu.cn (W.X.); 202321230101@std.uestc.edu.cn (W.Z.); 202322230103@std.uestc.edu.cn (W.W.)

**Keywords:** image restoration, image denoising, image deraining, image deburring, image desnowing, low-light enhancement

## Abstract

Although many deep learning-based image restoration networks have emerged in various image restoration tasks, most can only perform well in a specific type of restoration task and still face challenges in the general performance of image restoration. The fundamental reason for this problem is that different types of degradation require different frequency features, and the image needs to be adaptively reconstructed according to the characteristics of input degradation. At the same time, we noticed that the previous image restoration network ignored the reconstruction of the edge contour details of the image, resulting in unclear contours of the restored image. Therefore, we proposed an edge-aware guided adaptive frequency navigation network, EAFormer, which extracts edge information in the image by applying different edge detection operators and reconstructs the edge contour details of the image more accurately during the restoration process. The adaptive frequency navigation perceives different frequency components in the image and interactively participates in the subsequent restoration process with high- and low-frequency feature information, better retaining the global structural information of the image and making the restored image more visually coherent and realistic. We verified the versatility of EAFormer in five classic image restoration tasks, and many experimental results also show that our model has advanced performance.

## 1. Introduction

In digital image processing, image restoration is a crucial task that involves the reconstruction of damaged or missing parts, ensuring that the restored content is visually consistent with the original image. With the development of digital photography technology, people’s requirements for image quality are also increasing. However, in practical applications, due to the imperfect imaging system of the camera, unsatisfactory ambient lighting conditions, compression during transmission, and poor weather conditions that blur visual clarity, the images we obtain are often subject to varying degrees of degradation, such as noise interference, blur, compression artifacts, rain and fog interference, etc. These problems seriously affect the visual quality of the image and the performance of various visual tasks.

In recent years, deep learning technology has provided more possibilities for the development of image restoration technology. Among many previous explorations, generative adversarial networks [1,2] use adversarial training mechanisms to accurately fill in missing image information; multi-stage networks [3,4] sequentially and progressively optimize restoration effects; various types of convolutions [5,6] analyze image features from different dimensions; and attention mechanisms [7] focus on key areas to improve restoration accuracy. However, in-depth analysis shows that the network architecture and functional unit construction of such image restoration are mostly centered on spatial information mining. In actual restoration scenarios, the simple spatial domain strategy gradually becomes exhausted. It ignores the difference in frequency characteristics between clean images and degraded images, and it is difficult to accurately anchor the key restoration information contained in the frequency sub-bands, which hinders the restoration process. Some researchers found that different restoration tasks pay different attention to different frequency sub-bands [8]. For example, dark light enhancement and dehazing need more attention to low-frequency components. At the same time, the main difference between the target image and the degraded image for rain removal and deblurring is reflected in the mid-high-frequency components. Although researchers have previously noticed that better results can be achieved by utilizing low-frequency and high-frequency information in restoration tasks [9,10], they have not yet figured out how to enable the model to autonomously select different frequency components that are most suitable for the scenario under different tasks. To solve this problem, we explored general multi-task image restoration technology. During the experiment, we found that edge-filtering technology has shown great potential in many aspects of image restoration.

**Figure 1:**
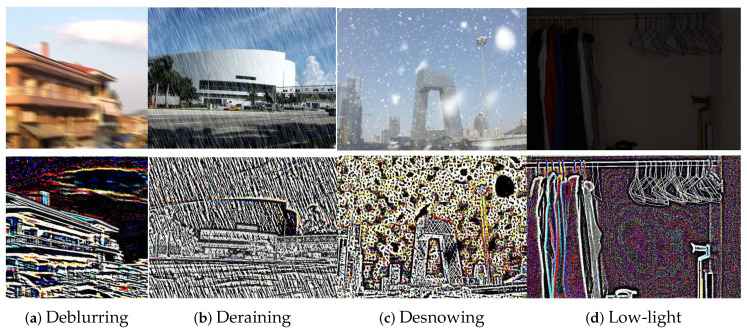
Edge information extraction.

As shown in Figure 1, even if affected by the four types of degradation shown above, edge filtering can effectively extract image boundary features, separate degradation factors, and retain the image texture structure, which is crucial for maintaining the details and clarity of the image after restoration. Using the image preprocessed by edge filtering as the input of each stage can obtain a restoration effect more consistent with visual perception than directly adding the degraded image to the restoration process [11,12].

**Figure 2:**
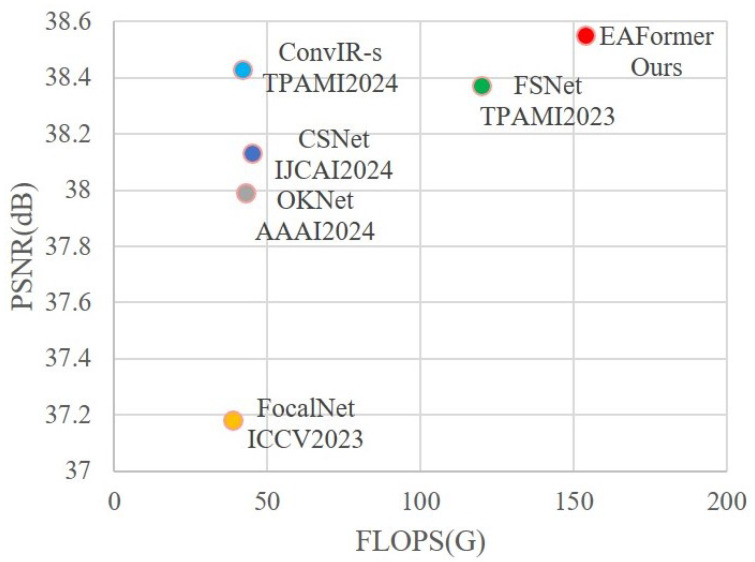
Our method outperforms the state-of-the-art image restoration models on the snow removal task, achieving a gain of 0.18 dB compared to FSNet [10].

Therefore, we propose EAFormer, a general image restoration network architecture that uses edge-aware prior information to guide adaptive frequency navigation. It can effectively combine the edge information and frequency characteristics of the image. The edge information focuses on the accurate restoration of local details and structures, while the frequency characteristics focus on the overall coherence of the image and the natural transition of texture. Specifically, we divide the network into three parts: encoder, bottleneck, and decoder. The encoder part is responsible for extracting the image’s feature representation and capturing the image’s multi-level information, including low-level texture information and high-level semantic information. With the assistance of the Dynamic Multi-edge Feature Extraction Filter (DMFEF), the encoder can pay more attention to the edge and structure information in the image, providing rich contextual features for subsequent modules. As the middle link of the network, the bottleneck part not only transmits the features extracted by the encoder but also integrates the edge-aware prior information. We introduce the Braided Dual-stream Channel Attention (BDCA) and the adaptive frequency navigation module (AFNM) at this stage. BDCA adopts a dual-stream structure, processing two different feature streams separately and capturing the features of different subspaces in parallel, which helps the model identify the importance of different regions in the image, thereby re-weighting the feature map and emphasizing those regions that are more critical to the final task. In order to limit the computational overhead, we only deploy this attention mechanism at the bottleneck. AFNM autonomously adjusts the frequency response of the feature map according to the incoming image information, strengthens essential structural information, and suppresses unnecessary noise and artifacts. The decoder part converts the high-level features extracted by the encoder and bottleneck parts back to the image space. We adopt an asymmetric decoder structure, combining the encoder features with the decoder features from coarse to fine from multiple scales through jump connections, which retains the image’s detailed information and enhances the image’s semantic consistency. Our goal is to build a robust model capable of achieving high-quality restoration in multiple scenarios. Therefore, we compared recent powerful image restoration models [9,10,13,14,15,16], and EAFormer achieved a relative lead in multiple datasets and restoration tasks. Taking the CSD [17] dataset for snow removal as an example, our model outperforms the state-of-the-art algorithms, as shown in Figure 2.

The main contributions of this work are as follows:

• We proposed an image restoration network, EAFormer, guided by edge information and autonomous selective frequency navigation, which can effectively focus on the local and overall features of the image and smoothly restore various degraded images.
• We designed an edge feature extraction filter with dynamic weight adjustment to achieve adaptive edge perception, making it more flexible in processing images with different textures and structures.
• We designed the Braided Dual-stream Channel Attention (BDCA) to independently learn features in different subspaces and reorganize and fuse them, which not only retains the advantages of each feature but also enhances the expressiveness of the features through interaction.
• We designed the adaptive frequency navigation module (AFNM), which decomposes the high- and low-frequency components of the image and adaptively modulates different degradation types, effectively improving the generalization performance of the model.
• All the above modules we designed are highly compatible and can be seamlessly integrated into any image restoration network architecture.

## 2. Related Work

### 2.1. CNN-Based Restoration Networks

CNN was introduced into the field of image restoration due to its success in image recognition tasks. CNN can automatically learn the hierarchical features of images, thereby achieving image restoration without manually designing features. CNN has been widely used in tasks such as denoising [5], deblurring [18], and deraining [19].

Most of the early CNN [20,21,22] image restoration models are based on non-blind image restoration, which means that they focus on restoring certain types of image damage without estimating the cause of image damage. For example, known blur kernel information is used to restore image clarity, or known noise type and noise level are used to achieve image denoising. This type of method is often limited in performance when facing unknown degradation types. DRUNet [23] combines U-Net and ResNet with a noise level map input to handle diverse noise levels via a single model, serving as an effective denoiser prior. Subsequently, CNN image restoration methods in blind scenarios [24,25] were derived, restoring images without knowing the cause or extent of damage, which is more challenging. In the field of blind denoising, DnCNN [5] was proposed to process images with unknown noise levels. These models no longer rely on fixed noise models but directly learn the characteristics of noise from data so that they can adapt to different noise levels and types. For blind deblurring, researchers have proposed models such as VBDeblurNet [26] and NR-IQA [27], along with DMPHN, [18] which employs a hierarchical multi-patch structure with residual learning for fine-to-coarse deblurring, and MIMO-UNet [28], which uses a single U-shaped network with a multi-input encoder, multi-output decoder, and asymmetric fusion to balance accuracy and efficiency. These models do not rely on pre-known blur kernels but restore images by learning end-to-end mapping from blurred to clear images. This approach requires the model to identify and compensate for various blur types, including motion blur and defocus blur. However, for a given degradation, a blind model may not perform better than a non-blind model. In relatively complex degradation patterns, such as irregular motion blur or non-uniform noise, the dataset of the blind model is more diverse, and the features are difficult to learn.

### 2.2. Transformer-Based Restoration Networks

The most significant advantage of Transformer over CNN is its self-attention mechanism, which can effectively capture long-range dependencies in the input sequence, which is of great significance for low-level visual tasks. In these tasks, the global information and local details of the image are equally important, and traditional CNNs may not be able to effectively utilize global information due to the limitations of their local receptive field.

In recent years, Transformer has also been widely used in image restoration tasks. Uformer [29] retains the U-shaped encoder–decoder structure. On the encoder side, it sequentially reduces the image space dimensions, just like accurately filtering information and extracting key features from massive image data; while in the decoder stage, it reverses the operation and gradually reshapes the image space dimensions to achieve fine restoration of image reconstruction. In each stage of the encoder and decoder, Uformer introduces a self-attention module, which enables the model to capture long-range dependencies of images at different scales and directly pass encoder features to the decoder through cross-scale connections, which helps to retain more detailed information during the restoration process. Another efficient image restoration model, SwinIR [30] adopts an encoder–decoder architecture and utilizes a shifted window multi-head self-attention mechanism. It divides the image window for self-attention calculation and shifts the interaction. It also performs feature fusion between the encoder and decoder and at different stages of the decoder, which can capture multi-scale features from low-level to high-level. Beyond the aforementioned models, other Transformer-based designs have also achieved notable performance in specific image restoration scenarios: DDABNet [31] is based on multi-supervision and hybrid attention, achieving excellent results in the field of image deblurring; Transfer CLIP [32] integrates noisy images and their multi-scale features from the frozen ResNet encoder of CLIP into the decoder, demonstrating better denoising performance; GRL [33] focuses on image features at multiple levels and shows good performance in the restoration of real-world images; Retinexformer [34] uses illumination representation to guide the restoration of low-light images under different illumination conditions, enabling better capture of long-range dependencies. However, with the increase in image resolution, the temporal and spatial complexity of the traditional self-attention mechanism increases quadratically, becoming a heavy burden on computing resources. In this context, Restormer [7] stood out and proposed the Multi-Dconv Head Transposed Attention mechanism, which cleverly reshaped the attention computing paradigm, significantly reduced computing overhead, successfully solved the problem of large image restoration, and brought image restoration quality to a new level. Although the current Transformer-based image restoration models have made significant progress in multiple restoration tasks, most of them learn features through supervised learning and need to transform to unsupervised or self-supervised learning in the future.

## 3. Method

In this section, we will give an overview of the image processing pipeline of EAFormer, followed by details of the Dynamic Multi-edge Feature Extraction Filter (DMFEF), Braided Dual-stream Channel Attention (BDCA), and adaptive frequency navigation module (AFNM).

### 3.1. Overall Pipeline

**Figure 3:**
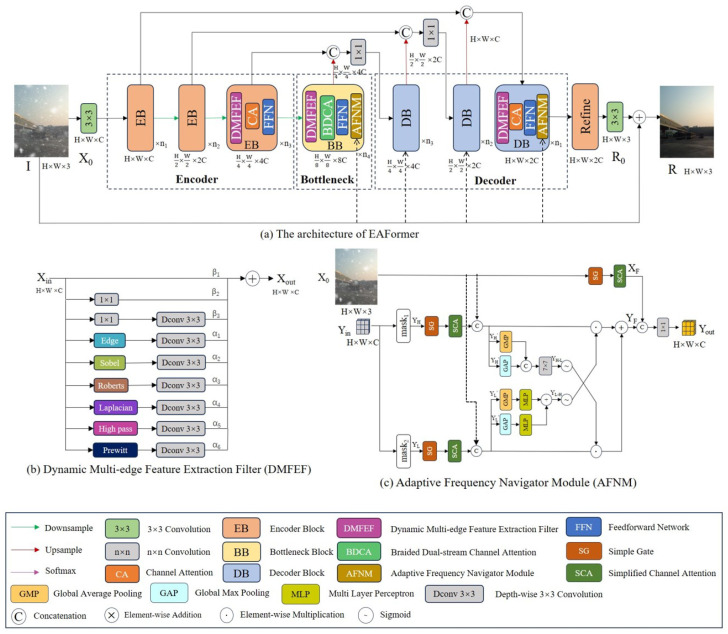
(**a**) Overview of the proposed EAFormer. The encoder–decoder architecture of the model uses the edge information of the DMFEF and the high- and low-frequency information of the AFNM to learn universal image restoration. (**b**) The DMFEF dynamically allocates the weights of convolution and edge operators on the path to filter out key information for guiding subsequent processing. (**c**) The AFNM decomposes the high- and low-frequency information of the input-degraded image and fuses the complementary information of different frequency components.

The EAFormer we proposed is an encoder–decoder architecture. The encoder module we designed consists of the DMFEF, channel attention, and a feedforward network. At the bottleneck, we replaced the channel attention with our designed BDCA. In order to find the most helpful feature combinations and frequency components for various tasks in the feature space, we added our designed AFNM at the end of the bottleneck module and the decoder module, as shown in Figure 3.

The input-degraded image $I\in R^{H\times W\times 3}$ first passes through a 3 × 3 convolution feature extractor to obtain $X_{0}\in R^{H\times W\times C}$, and then passes through each layer of the encoder and the downsampling layer to obtain the feature map $X_{i}\in R^{\frac{H}{2^{i}}\times \frac{W}{2^{i}}\times \frac{C}{2^{i}}}$, $i\in \left\{1,2,3\right\}$. The output of each layer of the encoder is jump-connected with the output of the decoder and upsampling operation at the corresponding position as the input of the decoder at the next position. This jump connection method is a cross-layer feature reconstruction, which realizes the information supplementation between layers and the enrichment of deep features in fusing feature maps at different levels. The image, after all decoders, passes through the feature refinement module, which uses the same structure as the encoder module, further refines the modeling, and applies 3 × 3 convolution to obtain the residual image $R_{0}$. $R_{0}$ superimposed on the degraded image I becomes the final repair result *R*:

(1) $$R=R_{0}+I$$

### 3.2. Dynamic Multi-Edge Feature Extraction Filter

Edge filters have been shown to have attractive performance in image restoration tasks such as image dehazing [35] and denoising [11], and edge feature extraction filters are more helpful in improving image feature expression in image restoration tasks than ordinary convolution [12]. In order to further explore the potential of filters in image restoration tasks, we design a dynamic multi-edge feature extraction filter (DMFEF) with general image restoration performance.

As shown in Figure 3b, we added ordinary convolution to ensure the essential performance of feature extraction. At the same time, the filter integrates a variety of classic edge detection operators, including Sobel, Roberts, Prewitt, and Laplacian operators. These operators are implemented as learnable convolution kernels to detect horizontal, vertical, and diagonal image edges. Specifically, by dynamically adjusting the weights of these operators, our model can adaptively optimize the feature extraction process according to different image content and degradation conditions. In order to improve the performance of the filter in the deblurring task, it is necessary to highlight and enhance the high-frequency information in the image, and we introduced a high-pass filter. The overall working performance process of the DMFEF is as follows:

(2) $$\begin{array}{cc} X_{\mathrm{out}}= & \alpha_{1}W_{d}^{E}X_{\mathrm{in}}+\alpha_{2}W_{d}^{S}X_{\mathrm{in}}+\alpha_{3}W_{d}^{R}X_{\mathrm{in}}+\alpha_{4}W_{d}^{L}X_{\mathrm{in}}+\alpha_{5}W_{d}^{H}X_{\mathrm{in}}\\ \; & +\alpha_{6}W_{d}^{P}X_{\mathrm{in}}+\beta_{1}X_{\mathrm{in}}+\beta_{2}W_{p}X_{\mathrm{in}}+\beta_{3}W_{d}W_{p}X_{\mathrm{in}} \end{array}$$

where $\alpha_{1}$ to $\alpha_{6}$ and $\beta_{1}$ to $\beta_{3}$ are the weights on each filter path, $W_{d}^{E}$, $W_{d}^{S}$, $W_{d}^{R}$, $W_{d}^{L}$, $W_{d}^{H}$, and $W_{d}^{P}$ represent the process of loading the parameters of Edge, Sobel, Roberts, Laplacion, Highpass, and Prewitt filters into 3 × 3 depth-wise separable convolution, $W_{p}\left(\;\cdot \;\right)$ represents 1 × 1 point-wise convolution, and $W_{d}\left(\;\cdot \;\right)$ represents 3 × 3 depth-wise separable convolution.

In Figure 1, we visualize the processing effect of the DMFEF in different restoration scenarios. The rain-stain image in Figure 1b has a high contrast at the edge of the fine line. After filtering, we can clearly see that the edge of the rain mark is significantly enhanced. This information will be passed to the subsequent rain removal stage so that the model can accurately locate and remove the degradation information. The rain removal dataset simulates the rain process by superimposing continuous scratches of almost uniform fineness and coarseness. In order to better simulate the camera’s “big in and small out” imaging principle and the characteristics of different snowflakes with different snow particle sizes, the snow removal dataset simulates the snowfall scene by superimposing masking patterns of different sizes and shapes with uneven distribution density on the original image, as shown in Figure 1c. These irregular characteristics are more difficult to capture than rain removal scenes, which thoroughly explains why previous standard image restoration models such as MPRNet [4], SwinIR [30], Uformer [29], Restormer [7], and NAFNet [36] have failed to achieve better performance in snow removal tasks. We use the feature information of the snow image after preprocessing with the DMFEF as prior knowledge and pass it into the subsequent model. We can see that the snow and non-snow areas are effectively separated. For the image taken under dark conditions in Figure 1d, which is seriously lacking in details and contrast, after filtering, we find that the local contrast of the low-contrast area is significantly improved.

### 3.3. Braided Dual-Stream Channel Attention

**Figure 4:**
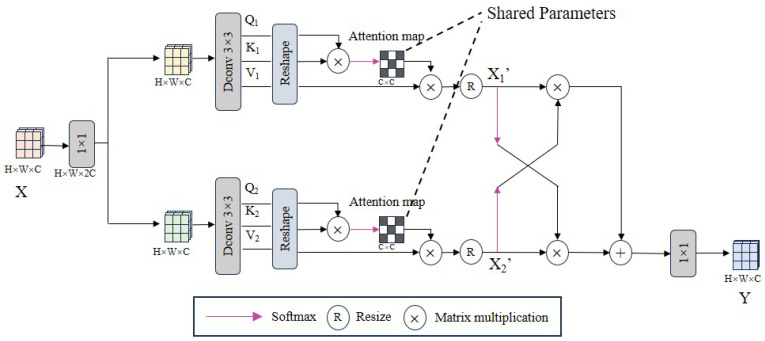
Information flow processing of BDCA.

The core component of the bottleneck module is the interleaved dual-stream channel attention mechanism (BDCA), as shown in Figure 4. The inspiration for our design of BDCA comes from the location of the bottleneck module at the junction of the encoder and the decoder. We simulate the subsequent interaction between the low-level image features from the encoder and the high-level image features from the decoder in the BDCA module, as shown in Figure 4.

Firstly, the input information stream $X\in R^{H\times W\times C}$ is first subjected to a 1 × 1 convolution to expand the channel capacity to twice the input. This operation helps the model capture richer feature representations and provides more space for subsequent feature interactions. Then, two independent paths are used to divert the information and map it channel by channel with a 3 × 3 depth-wise separable convolution so that each channel contains its query (*Q*), key (*K*), and value (*V*) projection:

(3) $$\begin{array}{cc} \; & Q_{i}=W_{d}^{Q_{i}}split\left(W_{p}^{Q_{i}}X\right)\\ \; & K_{i}=W_{d}^{K_{i}}split\left(W_{p}^{K_{i}}X\right)\\ \; & V_{i}=W_{d}^{V_{i}}split\left(W_{p}^{V_{i}}X\right),i\in \left\{1,2\right\} \end{array}$$

where split represents the process of diverting information from input X along two independent paths.

This split processing not only enhances the model’s ability to capture local features but also provides more detailed control for subsequent feature fusion. The $Q_{1}$, $K_{1}$ and $Q_{2}$, $K_{2}$ of the two channels are reconstructed and multiplied to obtain a shared attention map of size C × C to enhance relevant features and suppress irrelevant features. We do not calculate the attention maps of the two branches separately but adopt a shared attention map strategy. The reason is that the two branches are essentially homologous; that is, they come from the input *X*. Independent calculation of the attention map easily leads to the inability to effectively capture global context information, especially in scenes where background features or weather conditions cause pixel values to be similar but different from the background. This limitation hinders the model’s overall understanding of the image, especially in severe weather conditions such as rain, fog, or snow, which usually cause similar occlusion and brightness changes. Finally, by weighting $V_{1}$ and $V_{2}$ with the shared attention map, we obtain the adjusted $X_{1}^{\prime }$ and $X_{2}^{\prime }$, which is to dynamically adjust the importance of each channel feature according to the global context information and cross-channel correlation. After being processed by the softmax function, $X_{1}^{\prime }$ and $X_{2}^{\prime }$ can adjust each other’s feature contribution. This interaction helps the model capture and utilize the complementary information between the two channels, thereby achieving more effective feature fusion. The above process can be described as:

(4) $$\begin{array}{cc} \; & Y=W_{p}\left(X_{2}^{\prime }softmax\left(X_{1}^{\prime }\right)+X_{1}^{\prime }softmax\left(X_{2}^{\prime }\right)\right)\\ \; & X_{1}^{\prime }=V_{1}softmax\left(\delta \left(K_{1}\;\cdot \;Q_{1}/\alpha ,K_{2}\;\cdot \;Q_{2}/\beta \right)\right)\\ \; & X_{2}^{\prime }=V_{2}softmax\left(\delta \left(K_{1}\;\cdot \;Q_{1}/\alpha ,K_{2}\;\cdot \;Q_{2}/\beta \right)\right) \end{array}$$

where δ represents the process of sharing parameters in the attention graph, α and β are learnable parameters in the training process, which control the dot product size of $Q_{i}$ and $K_{i}$ on the two paths, respectively, $i\in \left\{1,2\right\}$.

### 3.4. Adaptive Frequency Navigation Module

The perception of the frequency of images under different degradation factors is different [8], so it is necessary to navigate the high- and low-frequency boundaries under different degradation conditions. Therefore, our design idea of AFNM is shown in Figure 3c. First, the input $Y_{in}\in R^{H\times W\times C}$ is converted from the spatial domain to the frequency domain inside $mask_{1}$ and $mask_{2}$, and then the high- and low-frequency boundaries of images under different degradation conditions are determined by the learnable spectrum mask size. The mask separates the frequencies, and the low-frequency components are mainly distributed in the center of the spectrum, while the high-frequency components are distributed on the periphery of the spectrum. Then, the high-frequency components outside the mask size and the low-frequency components within the mask size are restored to the spatial domain to obtain $Y_{H},Y_{L}\in R^{H\times W\times C}$. Specifically, this process can be defined as:

(5) $$\begin{array}{cc} \; & Y_{H}=ifft\left(fft\left(Y_{in}\right)⊙mask\right)\\ \; & Y_{L}=ifft\left(fft\left(Y_{in}\right)⊙\left(1-mask\right)\right) \end{array}$$

where ⊙ represents element-by-element multiplication, the mask is a filter that fills the periphery of the adaptive spectrum mask size with 0 elements to have the same shape as $fft(Y_{in})$, and fft/ifft represents fast Fourier transform/inverse transform.

Next, $Y_{H}$ and $Y_{L}$ are, respectively, mined through SimpleGate [36] and Simplified Channel Attention [36] to obtain $Y_{H}^{\prime },Y_{L}^{\prime }\in R^{H\times W\times C}$. Taking $Y_{H}^{\prime }$ as an example, the process is as follows:

(6) $$\begin{array}{cc} \; & Y_{H}^{\prime }=SCA\left(SG\left(W_{d}\left(W_{p}\left(Y_{H}\right)\right)\right)\right)\\ \; & SG\left(Y_{0}\right)=Y_{1}⊙Y_{2}\\ \; & SCA\left(Y\right)=Y⊙W_{p}\left(GAP\left(Y\right)\right) \end{array}$$

where GAP represents global average pooling, SG represents SimpleGate, and SCA represents Simplified Channel Attention and $Y_{1},Y_{2}\in R^{H\times W\times \frac{C}{2}}$ is obtained by splitting $Y_{0}\in R^{H\times W\times C}$ along the channel size.

Our processing mode for the processed high- and low-frequency features continues the interactive idea of BDCA to cross-realize bidirectional feature complementation. For the given $Y_{H}^{\prime }$ and $Y_{L}^{\prime }$, we apply global average pooling and global maximum pooling to them. For the branch of $Y_{H}^{\prime }$, the pooling results are concatenated in the channel dimension and passed to the 7 × 7 convolution. For the other branch of $Y_{L}^{\prime }$, the results of the two types of pooling are passed through the multi-layer perceptron, and the global average and maximum feature maps are nonlinearly transformed and added. Subsequently, the paths of $Y_{H}^{\prime }$ and $Y_{L}^{\prime }$ are subjected to sigmoid operations to generate a spatial gating map and a channel gating map between 0 and 1 to obtain $Y_{H-L}$ and $Y_{L-H}$, and $Y_{F}$ realizes the modulation of high- and low-frequency features:

(7) $$\begin{array}{c} Y_{F}=Y_{L}^{\prime }⊙Y_{H-L}+Y_{H}^{\prime }⊙Y_{L-H} \end{array}$$

Finally, the feature maps $X_{F}$ and $Y_{F}$ of the degraded image after SG and SCA adaptive adjustment are concatenated in the channel dimension and then subjected to 1x1 convolution dimensionality reduction processing to achieve feature fusion, and $Y_{out}\in R^{H\times W\times C}$ is obtained. The entire module maintains size consistency from input $Y_{in}$ to output $Y_{out}$:

(8) $$\begin{array}{c} Y_{out}=W_{p}\left(X_{F}concatY_{F}\right) \end{array}$$

## 4. Experiments

In this section, we use 16 datasets to verify the performance of EAFormer in 5 classic image restoration tasks: image deraining, image deblurring, image denoising, image desnowing, and low-light image enhancement. In order to highlight the statistical data of the experimental results, the highest performance indicators in the table are indicated in **bold**, and the indicators with the second lowest performance are underlined.

### 4.1. Implementation Details

We trained different models for different image restoration tasks. In all experiments, we used the following training parameters, as shown in Figure 3a, the number of encoder blocks is $n_{1}=4$, $n_{2}=6$, $n_{3}=6$, the number of bottleneck blocks is $n_{4}=8$, the number of decoder blocks is $n_{1}=4$, $n_{2}=6$, $n_{3}=6$, and the number of refinements is 4. The number of channels *C* is 48. We used the AdamW optimizer with $\beta_{1}=0.9$, $\beta_{2}=0.99$, and the learning rate varied from $3\times 10^{-4}$ to $1\times 10^{-6}$ through cosine annealing, and the weight decay parameter was set to $1\times 10^{-4}$. The loss function used is L1 loss:

(9) $$\begin{array}{c} Loss=\frac{1}{N}\sum_{i=0}^{N}∥X_{i}-Y_{i}∥ \end{array}$$

where N represents the number of pixels in the image, $X_{i}$ represents the i-th pixel of the restored image, and $Y_{i}$ represents the i-th pixel of the target image. We adopted a data augmentation strategy of horizontal and vertical flipping and trained the images in a progressive learning manner. As the number of training times increases, the patch size increases from 128 × 128 to 160 × 160 to 192 × 192 to 224 × 224 and finally to 256 × 256, with a total of 300 K iterations. Our model is trained on NVIDIA GeForce RTX 4090 and tested on NVIDIA Tesla A800.

### 4.2. Image Deraining Resultss

**Figure 5:**
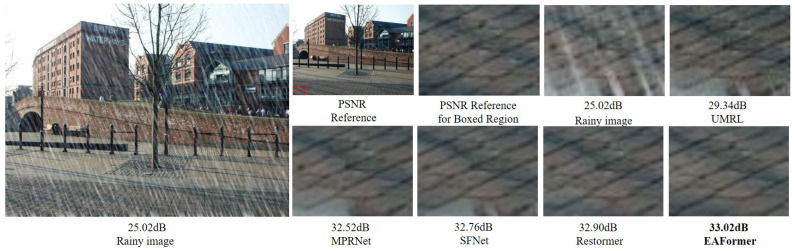
Image deraining results [4,7,9,19] on Test2800 [43].

For the image deraining task, we calculated the PSNR and SSIM metrics of the Y channel in the YCbCr color space and compared the performance with recent deraining models on the public datasets Test100 [37], Rain100L [38], and Test1200 [39]. The results in Table 1 show that the deraining performance of our proposed EAFormer is generally stronger than that of Restormer [7]. Figure 5 shows our deraining restoration example.

**Table 1:** We compare the deraining performance of general-purpose image restoration frameworks in recent years on the datasets Test100 [37], Rain100L [38], and Test1200 [39], with a focus on the PSNR [40] and SSIM [41,42] metrics.

Method	Test100 PSNR SSIM	Rain100L PSNR SSIM	Test1200 PSNR SSIM
UMRL [19]	24.41 0.829	29.18 0.923	30.55 0.910
MPRNet [4]	30.27 0.897	36.40 0.965	32.91 0.916
SFNet [9]	31.47 0.919	38.21 0.974	32.55 0.911
Restormer [7]	32.00 0.923	38.99 **0.978**	33.19 0.926
EAFormer (ours)	**32.02** **0.924**	**39.10** 0.977	**33.29** **0.927**

### 4.3. Image Deblurring Results

**Figure 6:**
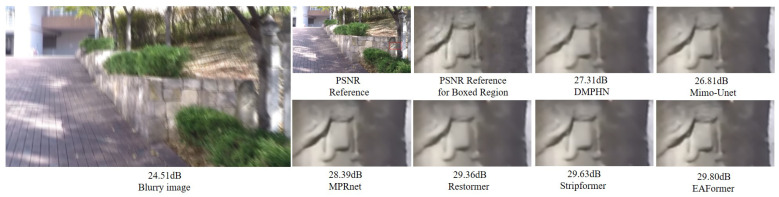
Single image motion deblurring results [4,7,18,28,49] on GoPro [44].

**Figure 7:**
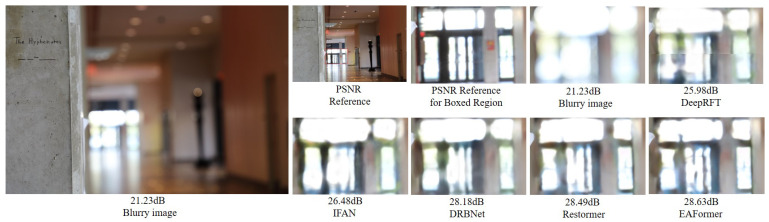
Defocus deblurring results [7,50,51,52] on DPDD [47].

In the image deblurring task, we evaluate the actual deblurring performance of EAFormer by two types of deblurring, including single image motion deblurring and defocus deblurring. We use two synthetic datasets, GoPro [44] and HIDE [45], and two real datasets, RealBlur-R [46] and RealBlur-J [46], to evaluate the performance of single image motion deblurring, and learn the restoration strategy of defocus deblurring based on the DPDD [47] dataset. We calculate the PSNR and SSIM indicators on three channels (if not explicitly stated in the following, the indicators are calculated on three channels by default). Here, we would like to point out that for the fairness of the experiment, our experimental results remove the trick of NAFNet [36] using the tlc [48] method to align the inconsistency between test and training. The results of single image motion deblurring and defocus deblurring are shown in Table 2 and Table 3. Figure 6 and Figure 7 visualize our method.

### 4.4. Image Denoising Results

**Figure 8:**
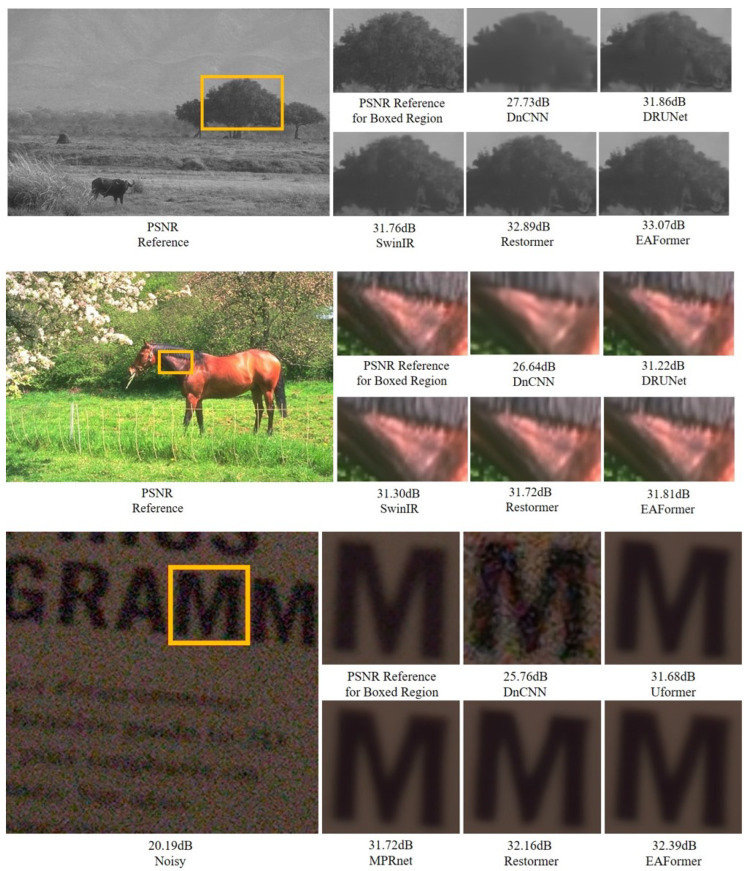
The first four rows show the results [5,7,23,30] of grayscale image denoising and color image denoising of BSD68 [53] and CBSD68 [53], respectively. The last two rows show the real image denoising examples [4,5,7,29] of SIDD [54].

In Table 4, Table 5 and Table 6, we trained a separate model for each noise level. We used it to verify the denoising performance of EAFormer on color images, grayscale images, and real images. Gaussian denoising was performed on color and grayscale images at $\sigma =\left\{15,25,50\right\}$, respectively. In terms of PSNR, in color image denoising on the CBSD68 [53] dataset, EAFormer outperforms the baseline model Restormer [7] by 0.05 dB when σ=25; in grayscale image denoising on the BSD68 [53] dataset, EAFormer leads Restormer [7] by 0.06 dB at σ=25; and in real image denoising on the SIDD [54] dataset, EAFormer is 0.07 dB better than Restormer [7]. To ensure the consistency of experimental conditions, the Restormer [7] was also progressively trained with patch sizes of 128, 160, 192, 224, and 256. The results show that our model has the best denoising level when σ=15. We visualized the denoising results of color, grayscale, and real images, as shown in Figure 8.

### 4.5. Image Desnowing Results

**Figure 9:**
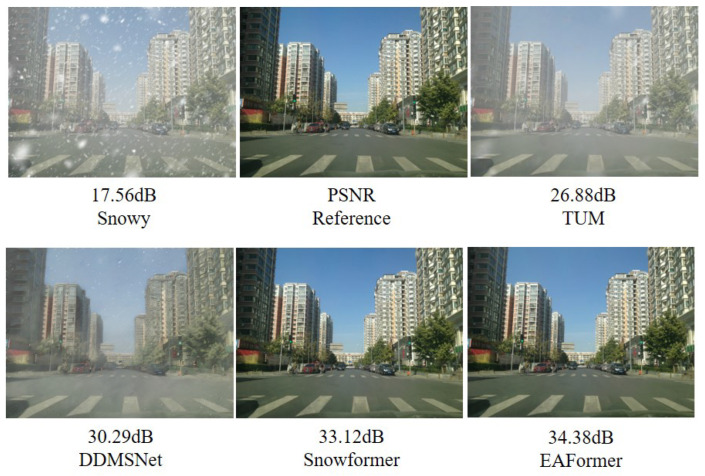
Desnowing results [60,61,62] on CSD [17].

We calculated the PSNR and SSIM indicators on the public snow removal dataset CSD2000 [17]. In Table 7, the results show that our model has significantly improved the performance of the snow removal task compared to the latest general-purpose repair model DSANet [58], and has increased by 0.12 dB in PSNR compared to the latest CONVIR-S [59] model. The visual results of snow removal repair are shown in Figure 9, where it can be observed that EAFormer achieves more realistic restoration results in the blue building compared to Snowformer [60], removing more of the shadows from the building.

### 4.6. Low-Light Enhancement Results

**Figure 10:**
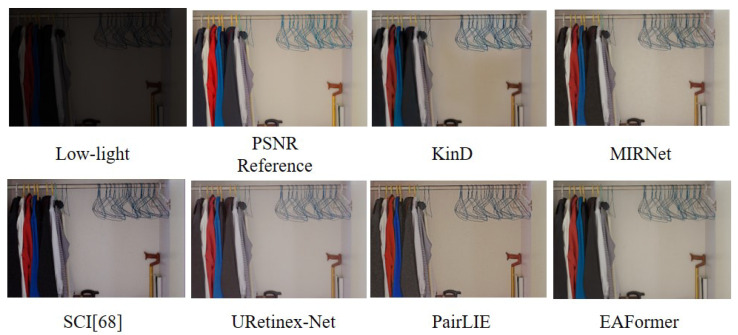
Low-light enhancement results [63,66,67,68,69] on LOL-V1 [64] and LOL-V2 [65].

We compared the existing state-of-the-art low-light enhancement model PairLIE [63] with the low-light datasets LOL-V1 [64] and LOL-V2 [65]. In Table 8, our model’s scores in PSNR and SSIM are significantly better than PairLIE [63]. As shown in Figure 10, we can find that KinD [66]-enhanced images have obvious dark areas and inaccurate clothing color recovery; MIRNet [67] lacks detail repair, with unclear clothing textures and blurry background walls; SCI [68] has issues with color fidelity, clothing color deviation, and overexposed areas; URetinex-Net [69] has uneven brightness adjustment and poor detail preservation; and PairLIE [63] has inaccurate color restoration and artificial appearance brightness enhancement. However, EAFormer achieved the most realistic low-light enhancement and was closest to the PSNR reference image. We can observe that the fine clothing texture and overall scene structure are well presented, and the brightness enhancement effect is uniform and natural, without excessive or insufficient local exposure. Under low-light conditions, the true color of the object is accurately restored, making the clothing color and background wall color in the enhanced image very close to the PSNR reference value.

### 4.7. Ablation Studies

In this section, all our ablation experiments are based on comparing rain removal and restoration results produced by Rain100L [38]. We verify the effectiveness of the proposed BDCA, AFNM, and DMFEF, and the results are shown in Table 9. In network (A), we remove the DMEFE from the encoder, bottleneck, decoder, and reconstruction modules of EAFormer to verify whether the edge information provided by the edge filter can guide the subsequent restoration process. In network (B), we replace the BDCA that only appears in the bottleneck with ordinary channel attention. In network (C), in order to test whether the AFNM can improve the overall performance of the model, we remove AFNM in the bottleneck and decoder and compare and verify whether the presence or absence of the AFNM affects the final restoration effect. Secondly, under the principle of a unified training strategy, we compare whether the modules with similar functions in other networks [8,11,12] are more substitutable than our modules and then more systematically verify the role of the proposed components. The results are shown in Table 10.

The results of the network (A) show that the performance of the model is affected without edge information as prior knowledge. This shows that edge information is indeed effective prior knowledge, and the model using edge information as prior knowledge can better capture the key structures in the image. Network (B) shows that our proposed BDCA is more adaptable to complex image features than ordinary channel attention, bringing a favorable gain of 0.04 dB. In network (C), the lack of adaptive frequency selection and modulation will limit the flexibility and effectiveness of the model in dealing with different types of image degradation. Removing the AFNM component will lead to a decrease in image restoration indicators.

In Table 10, we first compare the AFLB module with the frequency modulation function in [8], and we can see that our AFNM has a 0.10 dB PSNR performance improvement. We also compare the filter modules Adaptive Filter and ECB in [11,12]. The restoration performance of these filters applied to EAFormer is weaker than that of the DMFEF.

## 5. Limitations

We calculated the FLOPs and Params of each model when the input image size was uniformly 256 × 256, and also calculated the PSNR metric when processing the Rain100L [38], as shown in Table 11. It is not difficult to see that while our model achieves excellent performance across a variety of image restoration tasks, it also incurs a higher computational load and parameter count. However, it should be noted that for many practical applications, image restoration quality is often more critical than processing speed. For example, in fields such as medical image restoration and digital restoration of cultural relics, accurately restoring image details to ensure reliability for subsequent analysis or preservation takes precedence over processing efficiency. Therefore, for these tasks where quality takes precedence over speed, the trade-off between performance and computational cost achieved by the current model is acceptable. In the future, we will strive to further optimize the network architecture while maintaining the model’s current high performance, identify and remove redundant structures, and explore more efficient lightweight design solutions to reduce module size. This will reduce computational overhead while ensuring restoration quality, thereby improving the model’s practicality and generalization.

In addition, the data in Table 4 show that the denoising performance of our model is relatively limited when facing Gaussian noise with $\sigma =\left\{25,50\right\}$. We speculate that under the influence of high-intensity Gaussian noise, the edge and texture information of the image may be masked by the noise, making it difficult for the edge detection module to accurately identify and extract edge information. Inaccurate edge information will directly affect the subsequent image processing steps. On the other hand, our adaptive frequency navigation module is highly dependent on the accurate analysis of the frequency components of the image. Under the influence of such strong noise, it is difficult for the module to distinguish between signals and noise in the high-frequency area, resulting in a decrease in the actual denoising performance.

## 6. Conclusions

This paper proposes a general image restoration model with a multi-stage encoder-decoder architecture, which performs well in image deraining, deblurring, image denoising, image desnowing, and low-light image enhancement tasks. Our method introduces edge information to guide the decomposition of high-frequency and low-frequency components of subsequent images, which makes up for the shortcomings of traditional methods in edge preservation. When processing images with complex textures or blurred boundaries, it accurately extracts edge features to ensure that the restored image has clear contours and natural textures. At the same time, the adaptive frequency navigation module we designed can adaptively filter and modulate high-frequency and low-frequency boundaries according to different image degradation types, providing customized restoration strategies for diverse degraded images. Whether it is a low-light enhancement task that focuses on low-frequency information or a deraining and deblurring task that relies on mid- and high-frequency differential restoration, this module can accurately adjust the frequency response, enhance key structural information, and suppress noise and artifact interference to a certain extent, thereby ensuring that the restored image has excellent visual coherence and realism. For example, in low-light image enhancement, it can highlight the contours and details of the subject, and restore scene clarity and color saturation when removing rain. Finally, we rigorously verified the advancement and effectiveness of EAFormer through 16 datasets and five restoration tasks. Although EAFormer has performed well in multiple restoration tasks, we found that the designed model still encountered bottlenecks in improving the denoising performance of the model when faced with high-intensity noise. Despite its shortcomings, EAFormer has significant advantages in the field of cultural relic image restoration. It can accurately restore the delicate texture of cultural relics and enhance the clarity of structural details. In the future, we will strive to reduce the computing resource load while maintaining the stability of restoration accuracy, and create an efficient model that is adapted to edge computing resource constraint scenarios.

## Figures and Tables

**Table 2:** Image motion deblurring effects on GoPro [44], HIDE [45], RealBlur-R [46], and RealBlur-J [46].

Method	GoPro		HIDE		RealBlur-R		RealBlur-J	
	**PSNR**	**SSIM**	**PSNR**	**SSIM**	**PSNR**	**SSIM**	**PSNR**	**SSIM**
DMPHN [18]	31.20	0.942	28.94	0.915	34.02	0.907	27.79	0.841
Mimo-Unet [28]	30.99	0.937	28.79	0.911	33.81	0.898	27.62	0.832
MPRNet [4]	32.66	0.959	30.96	0.939	35.99	0.952	28.70	0.873
Restormer [7]	32.92	0.961	31.22	0.942	36.19	0.957	28.96	0.879
Stripformer [49]	33.08	0.960	31.03	0.940	35.99	0.952	28.69	0.870
EAFormer (ours)	**33.34**	**0.965**	**31.29**	**0.943**	**36.26**	**0.958**	**29.03**	**0.885**

**Table 3:** Comparison of defocus deblurring results on DPDD [47] dataset.

Method	Indoor Scenes				Outdoor Scenes				Combined			
	**PSNR ↑**	**SSIM ↑**	**MAE ↓**	**LPIPS ↓**	**PSNR ↑**	**SSIM ↑**	**MAE ↓**	**LPIPS ↓**	**PSNR ↑**	**SSIM ↑**	**MAE ↓**	**LPIPS ↓**
DeepRFT [50]	27.75	0.849	0.026	0.181	22.91	0.731	0.051	0.244	25.71	0.801	0.039	0.217
IFAN [51]	28.11	0.861	0.026	0.179	22.76	0.720	0.052	0.254	25.37	0.789	0.039	0.217
DRBNet [52]	28.49	0.872	0.025	0.166	22.99	0.735	0.051	0.229	25.72	0.811	0.038	0.183
Restormer [7]	28.87	0.882	0.025	0.145	23.24	0.743	0.050	0.209	25.98	0.811	0.038	0.178
EAFormer (ours)	**29.09**	**0.885**	**0.024**	**0.142**	**23.47**	**0.754**	**0.049**	**0.199**	**26.23**	**0.821**	**0.037**	**0.171**

**Note:** ↑ denotes the higher the value, the better; ↓ denotes the lower the value, the better.

**Table 4:** The PSNR indicators of grayscale image datasets set12 [5], BSD68 [53], and Urban100 [55] when $\sigma =\left\{15,25,50\right\}$.

Method	set12			BSD68			Urban100		
	**15**	**25**	**50**	**15**	**25**	**50**	**15**	**25**	**50**
DNCNN [5]	32.67	30.35	27.18	31.62	29.16	26.23	32.28	29.80	26.35
DRUNet [23]	33.25	30.94	27.90	31.91	29.48	26.59	33.44	31.11	27.96
SwinIR [30]	33.36	31.01	27.91	31.97	29.50	26.58	33.70	31.30	27.98
Restormer [7]	33.34	30.99	**27.99**	31.92	29.49	26.56	33.63	31.36	**28.33**
EAFormer (ours)	**33.40**	**31.05**	27.97	**31.98**	**29.53**	**26.63**	**33.80**	**31.42**	28.19

**Table 5:** The PSNR indicators of the color image datasets CBSD68 [53], Kodak24 [56], McMaster [57], and Urban100 [55] are as follows; our model shows an overall improvement over the baseline denoising model Restormer [7] when σ=15 and σ=25.

Method	CBSD68			Kodak24			McMaster			Urban100		
	**15**	**25**	**50**	**15**	**25**	**50**	**15**	**25**	**50**	**15**	**25**	**50**
DnCNN [5]	33.90	31.24	27.95	34.60	32.14	28.95	33.45	31.52	28.62	32.98	30.81	27.59
DRUNet [23]	34.30	31.69	28.51	35.31	32.89	29.86	35.40	33.14	30.08	34.81	32.60	29.61
SwinIR [30]	34.39	31.78	28.56	35.34	32.89	29.79	**35.61**	33.30	30.30	35.10	32.88	29.82
Restormer [7]	34.38	31.77	**28.57**	35.44	32.96	29.96	35.51	33.28	30.18	35.06	32.86	**30.00**
EAFormer (ours)	**34.43**	**31.82**	28.55	**35.49**	**33.03**	**30.02**	35.60	**33.34**	**30.33**	**35.13**	**32.95**	29.96

**Table 6:** We performed real image denoising on the SIDD [54] dataset and found that our model achieved a PSNR gain of 0.07 over the state-of-the-art real image denoising model Restormer [7].

Method	SIDD
DnCNN [5]	23.66
Uformer [29]	39.77
MPRNet [4]	39.71
Restormer [7]	39.99
EAFormer (ours)	**40.06**

**Table 7:** Comparison of snow removal results in CSD2000 [17].

Method	CSD2000	
	**PSNR**	**SSIM**
DDMSNet [61]	34.25	0.961
TUM [62]	30.10	0.933
Snowformer [60]	37.43	0.984
OKNet [13]	37.99	0.990
DSANet [58]	38.09	0.990
FSNet [10]	38.37	0.991
CONVIR-S [59]	38.43	0.991
EAFormer (ours)	**38.55**	**0.992**

**Table 8:** Low-light enhancement results in LOL-V1 [64] and LOL-V2 [65].

Method	LOL-V1		LOL-V2	
	**PSNR**	**SSIM**	**PSNR**	**SSIM**
KinD [66]	15.65	0.688	17.01	0.717
MIRNet [67]	16.37	0.699	17.64	0.731
SCI [68]	21.76	0.775	24.65	0.802
URetinex-Net [69]	22.44	0.800	26.89	0.846
PairLIE [63]	23.59	0.848	27.40	0.859
EAFormer (ours)	**24.00**	**0.861**	**27.77**	**0.870**

**Table 9:** Ablation studies of each module.

Network	DMFEF	BDCA	AFNM	PSNR	SSIM
(A)		✓	✓	38.77	0.974
(B)	✓		✓	39.06	0.977
(C)	✓	✓		38.63	0.973
EAFormer	✓	✓	✓	**39.10**	**0.977**

**Note:** ✓ denotes that the corresponding module is used in the network.

**Table 10:** We define A→B to only represent the process of replacing module A with module B. The other modules are consistent with the structure of EAFormer. This table shows the indicators after module replacement.

Network	PSNR	SSIM
AFNM→AFLB [8]	39.00	0.976
DMFEF→Aaptive Filter [11]	39.04	0.976
DMFEF→ ECB [12]	38.79	0.974
EAFormer	**39.10**	**0.977**

**Table 11:** Comparison of performance of different networks with Flops and Params.

Network	PSNR	SSIM	Flops (G)	Params (M)
NAFNet [36]	36.96	0.971	63.33	67.80
Restormer [7]	38.99	0.978	140.99	26.13
SFNet [9]	38.21	0.974	125.43	13.27
X-Restormer [70]	39.10	0.978	164.30	26.03
EAFormer	39.10	0.977	154.59	35.87

## Data Availability

Our code is available at https://github.com/shdjak/xwj-EAFormer (accessed on 11 September 2025).
